# Impact of the inclination angle of corrugated energy absorbers on the crashworthiness performance: numerical study

**DOI:** 10.1038/s41598-026-45983-3

**Published:** 2026-04-07

**Authors:** Mahmoud Sheikh Sobeh, Abdel-Hamid I. Mourad, Mohammad A. AlKhedher, Mallik Sudhanshu

**Affiliations:** 1 Mechanical and Aerospace Engineering Department, United Arab Emirates University, Al-Ain, P.O. Box 15551, UAE; 2 National Water and Energy Center, United Arab Emirates University, Al-Ain, P.O. Box 15551, UAE; 3 Mechanical and Industrial Engineering Department, Abu Dhabi University, Abu Dhabi, P.O. Box 59911, UAE; 4 Department of Metallurgical Engineering and Materials Science, Indian Institute of Technology Bombay, Mumbai, P.O. 400076, India

**Keywords:** Corrugated tube, Total absorbed energy, Mean crushing load, Stroke efficiency, Axial loading, ANSYS/explicit dynamics, Corrugation, Engineering, Materials science

## Abstract

In the present study, cylindrical energy absorbers with various corrugation inclination angles were numerically analysed to evaluate their crashworthiness under quasi-static axial loading. Simulations were performed using ANSYS/Explicit Dynamics, operated in a quasi-static regime by applying a sufficiently slow loading rate to suppress inertial effects. Seven designs were evaluated: a smooth tube (no corrugation) and corrugated tubes with inclination angles of 0°, 15°, 30°, 45°, 60°, and 90°, measured from the horizontal axis. The total absorbed energy (TAE), mean crushing load (MCL), stroke efficiency (SE), specific energy absorption (SEA), initial peak force (IPF), and crushing force efficiency (CFE) were computed for each inclination case. TAE was calculated by exporting the force–displacement data from ANSYS as tabulated results, importing them into Excel sheet, and applying the trapezoidal rule to estimate the area under the curve. The case of 90° recorded the highest TAE (8,382.55 J), followed by the smooth (non-corrugated) tube (6,049.32 J). The lowest TAE value was the case of 30° (4,278.42 J) for 30°, indicating that mid-range inclination angles (30° and 45°) may lead to early instability and reduced plastic fold development. The 60° and 90° configurations showed improved performance, likely due to the formation of diagonal or shear-assisted folding paths that spread deformation more uniformly and delayed densification. The initial peak force (IPF) is lowest for the 0° corrugated case (27,555 N) and highest for the 90° case (102,570 N). This trend aligns with shell structure mechanics and plastic collapse theory, which suggest that as the corrugation angle increases, the structure develops more transverse stiffness and localized geometric resistance. These features delay the onset of plastic deformation and require a higher initial load to initiate global collapse. As a result, the load path shifts from a mostly axial mode to a combined axial-transverse response. This transition leads to higher initial resistance and explains the rise in peak force with steeper corrugation angles. This study provides an insight into the impact of corrugations and corrugation angle on the energy absorber performance, and crashworthiness systems in different applications (e.g. automotive and aerospace engineering, structural).

## Introduction

Crashworthiness, defined as a structure’s ability to withstand impact stresses, has been extensively studied over the past two decades, emphasizing the need for energy absorbers that prevent sudden failure by distributing impact energy through controlled deformation^1^.

Across the extensive literature on thin-walled structures for crashworthiness applications, several consistent findings emerge regarding their geometry, materials, and design strategies. Thin-walled tubes are widely acknowledged for their lightweight nature, manufacturability, and excellent ability to dissipate impact energy through stable and progressive plastic deformation^2–4^. Geometric optimization plays a critical role, with circular, hexagonal, and octagonal cross-sections exhibiting higher crushing resistance than rectangular or square designs, while multi-cell and branched configurations further enhance energy absorption by increasing load paths and deformation zones^5–7^. Material innovation also drives performance improvements; polymer matrix composites and fibre-reinforced tubes provide higher specific energy absorption (SEA) than traditional metals and enable tailored failure mechanisms^4,8–10^. Hybrid designs, including foam-filled tubes, honeycomb cores, and auxetic metamaterials, show significant enhancement in specific energy absorption (SEA) and energy dissipation efficiency due to internal support structures and controlled collapse behaviours^11–14^. Studies consistently emphasize predictable deformation modes such as local buckling, wall bending, and progressive folding as critical for effective crashworthiness, and confirm the reliability of finite element models in simulating these behaviours when validated with experimental results^2,9,15–18^. Key performance indicators like SEA, crush force efficiency, and stroke efficiency are widely used to evaluate and optimize absorber designs under both static and dynamic loading conditions^5,17^. The use of lattice structures with gradient densities, multi-layered composites, and vertically cut honeycombs further expands the design potential for reusable, high-performance energy absorbers, particularly in automotive, aerospace, and public transportation sectors^3,13,18,19^. Collectively, these findings underscore the importance of material-geometric synergy and informed numerical modelling in developing next-generation energy-absorbing systems aimed at minimizing occupant injury and structural damage during impact events.

To improve crash behaviour, additional radiation absorbers can be attached to thin-walled tubes designed to dissipate impact energy. Materials that reversibly absorb energy, such as pneumatic dashpots or elastomeric shock absorbers, dissipate the energy collected through elastoplastic or ductile deformation modes^8^. In practical scenarios, energy absorption predominantly occurs via plastic deformation. Consequently, thin-walled metal tubes, such as those fabricated from low-carbon steel or anodized alloys, have been extensively studied, especially with the inclusion of fillers to enhance performance. Polymethyl cellulose (PMC) fibre composites exhibit brittleness and dissipate energy through inversion and fracture mechanisms. Typical collapse behaviours of composite fibre materials include sequential crushing and crack propagation^20^. Previous crash protection studies on nanocomposite energy-absorbing tubes have examined factors such as cross-sectional geometry, fabrication methods, fibre content, laminate orientation, triggering mechanisms, and plate thickness each influencing peak load resistance and post-yield stiffness.

In response to the increasing demand for safety in transportation, several innovative energy-absorbing devices have been developed. Layered composite structures combining crochet-sintered mesh tubes with thin-walled tubes demonstrated excellent axial crush resistance and enhanced energy absorption. Increasing the number of layers further improved performance, with the primary failure mode governed by the thin-walled tube^21^. These findings suggest the viability of such layered systems in automotive and aerospace crash applications.

Thin-walled tubular structures are commonly employed in vehicles trains, ships, and aircraft for their ability to dissipate impact energy and safeguard both passengers and infrastructure. These structures are engineered to undergo controlled elastic and plastic deformation to maximize force attenuation^22^. Extensive studies have evaluated tubes of various cross-sectional shapes including cylindrical, trapezoidal, rectangular, polygonal, hexagonal, and octagonal aiming to optimize load transfer and energy absorption. However, limitations in manufacturing processes have constrained further improvements through geometrical modifications alone^23^.

Recent advancements have focused on combining metal tubes with alternative materials to form hybrid composites, significantly improving crashworthiness. Three principal composite types have emerged: filled composites, stacked polycaprolactone structures, and cross-bonded solid-core tubes. These configurations demonstrate enhanced mechanical responses under impact, with promising applicability in energy absorption systems across transportation and defence sectors^24^.

Thin-walled tubes, favoured in aerospace and automotive sectors for their high strength to weight ratios, exhibit enhanced energy absorption through gradual elastic and plastic deformation modes and have been the focus of numerous quasi-static and dynamic load investigations^25^. Strategies to improve crash resistance include using multiple tubes, corrugated or curved surfaces, and filling tubes with metals, synthetic foams, biological materials, or honeycombs, which significantly boost energy absorption and structural safety. Notably, the radial distance between concentric tubes influences their absorption capacity, with closer tubes absorbing more energy, particularly under dynamic loading^25^. Designing safe and high performance transport products remains a critical challenge, as highlighted in studies like^26^, which demonstrated that foam-filled ultralight structures improve energy absorption as foam density increases and thickness decreases, outperforming solid structures^26^. Composite energy absorption tubes, incorporating materials such as cellulose fibres or synthetic fibre-reinforced thermoplastics, continue to gain importance due to their hybrid mechanical properties and enhanced crashworthiness^27^. Bio-inspired designs derived from biological systems offer superior energy absorption compared to traditional counterparts and have been developed using various materials and manufacturing methods including 3D printing^28–31^. Corrugated tubes, in particular, outperform smooth tubes of equivalent size by exhibiting greater energy absorption, more stable deformation, and sensitivity to corrugation depth and pitch^31^. The transportation industry’s rapid growth has led to numerous innovations in energy absorbers, with metal thin-walled tubes and epoxy composites commonly used in vehicles such as cars, trains, ships, and helicopters to safely absorb and dissipate impact forces via regulated folding and hinging during collapse^32^. While numerous tube shapes have been examined including cylindrical, trapezoidal, polygonal, and tapered variants further enhancement of energy absorption via cross-sectional shape alteration is constrained by manufacturing limitations^32^. Composite tubes combining metal and other materials have shown promising crashworthiness, with classifications including filled composites, layered configurations, and cross-joined cylinders^33^. Numerical studies on tapered thin-walled tubes indicate that greater taper angles and larger impact-end diameters lead to higher peak forces and energy absorption, underscoring their applicability in crashworthy designs.

Theoretical frameworks such as plastic collapse theory and shell structure mechanics provide essential context for understanding how geometry influences the energy absorption behaviour of thin-walled cylindrical structures. According to plastic collapse theory, energy dissipation during axial compression primarily occurs through the formation and propagation of plastic hinges, with geometry playing a key role in determining their location, number, and contribution to overall deformation^34,35^. Shell structure mechanics, on the other hand, examines how curved thin-walled components behave under compressive loading, with particular focus on how axial stiffness, local buckling, and geometric reinforcement interact to influence stability and collapse modes^36,37^.

Although many studies have focused on improving the crashworthiness of thin-walled energy absorbers, some important aspects still need more attention. Researchers have explored a variety of parameters, including different materials, cross-sectional shapes, and filler types like foam or honeycomb. These have led to better energy absorption and more stable deformation. However, one specific factor the inclination angle of corrugations has not been studied in depth. While some work has looked at corrugation patterns like depth or spacing, very few have examined how the angle at which the corrugation is oriented affects the tube’s ability to absorb energy during impact. This is a notable gap, especially considering how much this angle could influence deformation paths, folding behaviour, and stress distribution. To date, no study has systematically analysed how changing the corrugation inclination angle affects key crashworthiness measures like energy absorption or peak force under the same conditions. Because of this, the main aim of this research is to fill that gap by using numerical simulations to study the effect of corrugation angle on the crash performance of cylindrical energy absorbers. Key indicators evaluated were Initial Peak Force (IPF), Mean Crushing Force (MCF), Total Absorbed Energy (TAE), and Specific Energy Absorption (SEA).

## Materials and methods

This study employed a simulation-based approach to investigate the effect of corrugation inclination angles (0°, 15°, 30°, 45°, 60°, and 90°) on the crash performance of thin-walled cylindrical aluminium tubes. The analysis was conducted in ANSYS Explicit Dynamics to examine deformation, energy absorption, and force transmission under axial quasi-static loading. The reference study did not specify the aluminium grade; however, the reported material properties and stress–strain curve were directly used to define the material model.

**Fig. 1:**
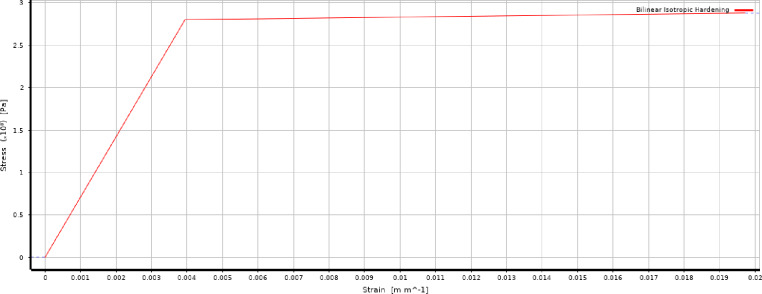
aluminum bilinear isotropic hardening data chart.

All models had identical geometric dimensions, including tube length, diameter, wall thickness, corrugation depth, and spacing, ensuring that only the inclination angle varied. Material properties were obtained from published experimental work^32^, which also served for validation. A 3D model was used to capture wall thickness and plastic deformation effects, with a bilinear isotropic hardening model representing aluminium behaviour. The material was defined with a Young’s modulus of 7.1 × 10^10 Pa, Poisson’s ratio of 0.33, bulk modulus of 6.9608 × 10^10 Pa, and shear modulus of 2.6692 × 10^10 Pa (Fig. 1).

The simulation setup used bonded contacts at the tube ends, frictionless self-contact to allow folding, and a rigid plate to apply compression. The mesh consisted of refined tetrahedral elements, adjusted to balance accuracy and computational cost. Custom time step settings and mass scaling were applied for stability. One tube end was fully fixed, while the rigid plate applied a constant-speed downward displacement. Outputs included force–displacement curves, energy absorption, and deformation modes.

Model validation was performed by replicating the experimental setup in^32^, which used a loading rate of 5 mm/min (0.083 mm/s), consistent with quasi-static conditions where inertial and strain rate effects are negligible. The numerical results showed close agreement with the experimental data, confirming the model’s accuracy. Following validation, simulations were extended to the inclined corrugated geometries.

## Results and discussions

To validate the numerical model, ANSYS Explicit Dynamics simulation results were compared with a published experimental study on corrugated cylindrical energy absorbers. A reference configuration from the literature was reproduced, and key crashworthiness indicators Total Absorbed Energy (TAE), Mean Crushing Load (MCL), and Stroke Efficiency (SE) were used for comparison. Percentage errors quantified the agreement between simulation and experiment.

Some idealizations were applied in the model. Boundary conditions were fixed, and material properties were assumed uniform to reduce computational complexity and ensure stability given limited processing resources. These simplifications differ from physical tests, where manufacturing imperfections and material variability occur, but the results showed strong correlation with experimental data, confirming the model captures the core mechanical behaviour. This validation supports proceeding to study the effect of varying corrugation inclination angles.

Table 1 summarizes the crashworthiness parameters used to evaluate the energy absorber models: TAE, MCL, Specific Energy Absorption (SEA), Initial Peak Force (IPF), and Crushing Force Efficiency (CFE). Each parameter provides a specific measure of performance under axial loading.

**Table 1:** Crashworthiness parameters summary.

Parameter	Definition	Higher Value Means	Is Higher Better?
Total Absorbed Energy	Total energy absorbed during deformation (usually in Joules)	The structure can take more energy before failure	Yes, better energy absorption.
Mean Crushing Load	Average force sustained during the crushing process	Stronger structural resistance over the stroke	Generally yes, but balance is needed
Specific Energy Absorption (SEA)	Absorbed energy per unit mass (J/kg)	More energy absorption efficiency	Yes, higher efficiency with less weight
Initial Peak Force	The first peak force when impact begins	High spike at the start of crash	No, high peak force = more risk to occupants
Mean Force	Average force during the entire crushing process	Stronger average force	Yes, contributes to energy absorption
Crushing Force Efficiency (CFE)	Ratio of mean force to peak force:$\:\frac{{F}_{mean}}{{F}_{peak}}$	How steady the crushing is	Yes, higher CFE = smoother, safer energy dissipation

### Model-by-model discussion for modeling previous experimental study

In the experimental study by Eyvazian^32^, a series of aluminum tubes with varying geometries and corrugation patterns were tested under quasi-static axial loading to investigate their crushing behavior and energy absorption performance. Each specimen was assigned a unique identifier that reflects its geometric type and wall thickness. For instance, S101 and S151 represent simple (non-corrugated) circular tubes with wall thicknesses of 1.0 mm and 1.5 mm, respectively, and serve as baseline references for comparison^32^. The labels CD10D and CD15D denote axially corrugated tubes fabricated with the same respective wall thicknesses where the corrugation depth and length were controlled using a custom stamping method designed to enforce symmetric folding^32^. Tubes labeled TF10C and TF15D correspond to parallel corrugated designs, where two identical tubes were phase-shifted by half a wavelength to enhance load uniformity^32^. Meanwhile, T10BF and T15BH are laterally corrugated tubes, with circular corrugations formed around the circumference, resulting in higher initial stiffness and altered collapse behavior^32^. The CM15 specimen incorporates variable corrugation depths along its length, offering a progressive response to increasing impact loads^32^. All the corrugated specimens were manufactured using a specialized stamping process involving rotating steel dies, offering significant flexibility in corrugation geometry while maintaining cost-effectiveness^32^. A copy of the specimen details is illustrated in Table 2 below. All the specimens were modeled numerically using refined tetrahedral elements to capture local deformations accurately. The simulations were executed on limited hardware, which resulted in long computational times due to the fine mesh and the complexity of the nonlinear crushing behavior. In this section, a detailed numerical re-analysis of these configurations is presented, comparing the original experimental results with the simulated outputs to evaluate the fidelity and predictive accuracy of the numerical approach.

**Table 2:** The specimens’ dimensions and summary of the previous experimental study test results^32^.

Specimen no.	Thickness (mm)	Diameter (mm)	Corrugation amplitude (mm)	Corrugation length (mm)	Mean load (*N*)	Total energy absorbed (J)	Stroke efficiency (%)
S101	1	77.2	-	-	9.05E + 03	4.69E + 05	0.77
S102	1	77.2	-	-	7.26E + 03	3.80E + 05	0.76
Diamond							
S151	1.5	78.4	-	-	1.51E + 04	7.93E + 05	0.73
S152	1.5	78.4	-	-	1.61E + 04	8.31E + 05	0.72
CD10D	1	77.2	2.2	13.5	6.80E + 03	3.35E + 05	0.71
CD15D	1.5	78.4	2.4	13.5	1.24E + 04	6.17E + 05	0.71
TF10C	1	77.2	2.5	13.5	1.41E + 04	7.58E + 05	0.71
TF15D	1.5	78.4	2.7	11.8	2.30E + 04	1.02E + 06	0.74
CM15	1.5	78.4	2.9	13.84	1.42E + 04	6.25E + 05	0.63
			2.3	13.84			
			1.8	13.84			
			1.3	13.84			
T10BF	1	77.2	1.8	7	1.05E + 04	4.36E + 05	0.74
T15BH	1.5	78.4	1.8	7	2.00E + 04	1.07E + 06	0.77

#### S101 specimen

**Fig. 2:**
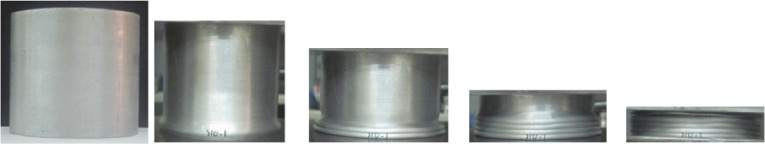
Different stages of experimental axial collapse of specimens S101 specimen^32^.

**Fig. 3:**
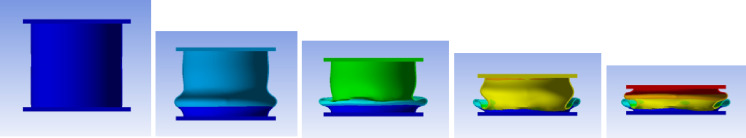
Different stages of axial collapse of specimens S101 simulation model.

**Fig. 4:**
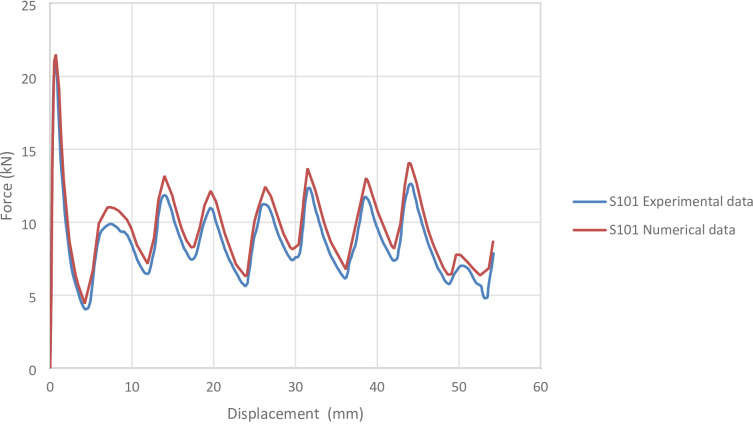
Force–displacement curve for the simulated and experimental results of S101 specimen and model.

Figures 2 and 3 present the axial collapse stages of the S101 specimen in both experiment and simulation. Experimentally, the total absorbed energy was 469.00 J, the mean crushing load was 9050.00 N, and the stroke efficiency was 0.77. The numerical model produced values of 468.48 J, 8640.90 N, and 0.68, with percentage errors of 0.11%, 4.52%, and 11.43%, respectively. The corresponding force–displacement curves of the numerical and experimental data are shown in Fig. 4.

The model was constructed with 63,466 elements, providing a refined mesh that accurately captured the progressive deformation under axial compression. The underprediction of stroke efficiency is attributed to the high sensitivity of this parameter to geometric imperfections, plastic folding, and material variability, which are idealized in finite element models. Despite this, the agreement in absorbed energy and load response demonstrates that the S101 simulation is reliable and serves as a valid reference for numerical validation.

#### S151 specimen

**Fig. 5:**
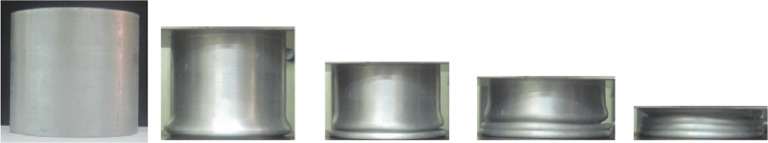
Different stages of experimental axial collapse of S151 specimen^32^.

**Fig. 6:**
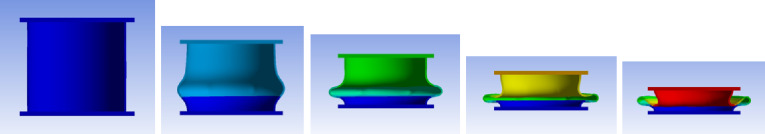
Different stages of axial collapse of specimens S151 simulation model.

**Fig. 7:**
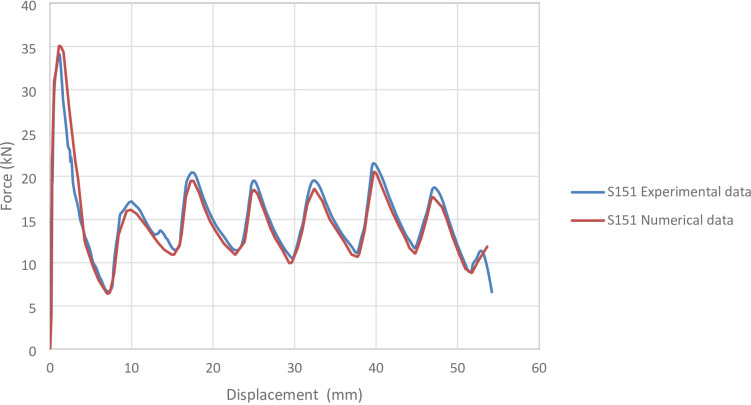
Force–displacement curve for the simulated and experimental results of S151 specimen and model.

Figure 5 presents the axial collapse sequence of the S151 experimental specimen, while Fig. 6 shows the corresponding simulation stages. The experimental results reported an absorbed energy of 793.00 J, a mean crushing load of 15,100.00 N, and a stroke efficiency of 0.73. In comparison, the simulation predicted an absorbed energy of 811.33 J (2.31% higher), a mean crushing load of 14,964.68 N (0.90% lower), and a stroke efficiency of 0.68 (6.58% lower). The simulated and experimental force–displacement curve, shown in Fig. 7, presents the corresponding force–displacement curves for the numerical and experimental data. The model was discretized using 64,035 elements, providing a detailed representation of the axial response. The slight underestimation in stroke efficiency is attributed to the idealized numerical conditions. Overall, the simulation of the S151 specimen showed good agreement with the experimental data, particularly for energy absorption and mean load.

#### CD10D specimen

**Fig. 8:**
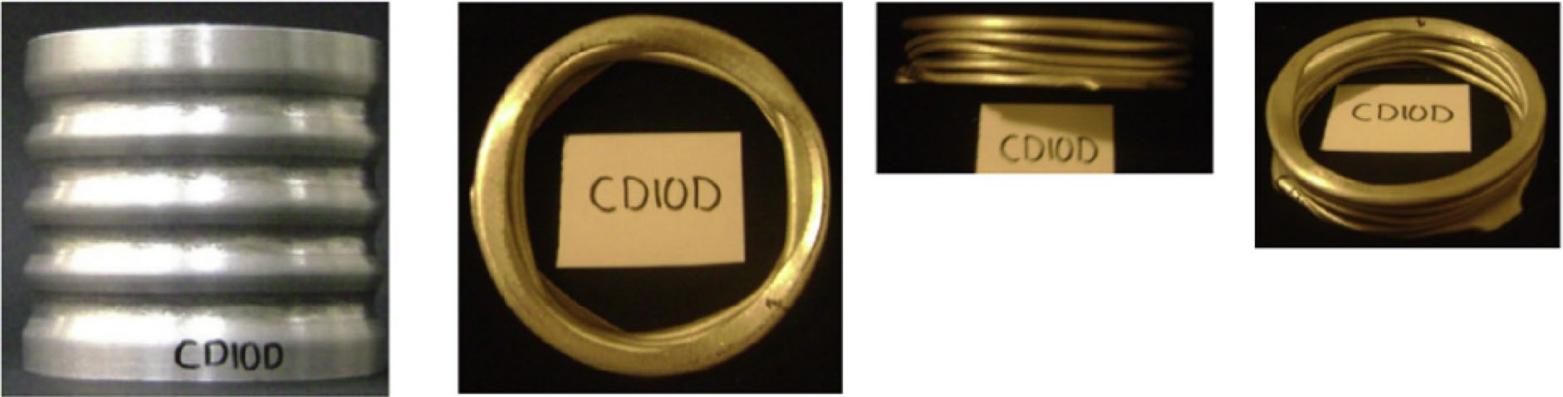
Different stages of experimental axial collapse of CD10D specimen^32^.

**Fig. 9:**
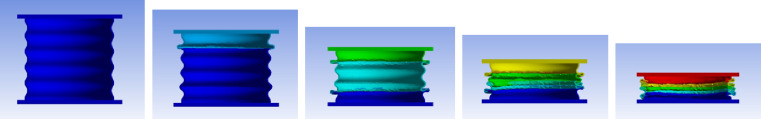
Different stages of axial collapse of specimens CD10D simulation model.

**Fig. 10:**
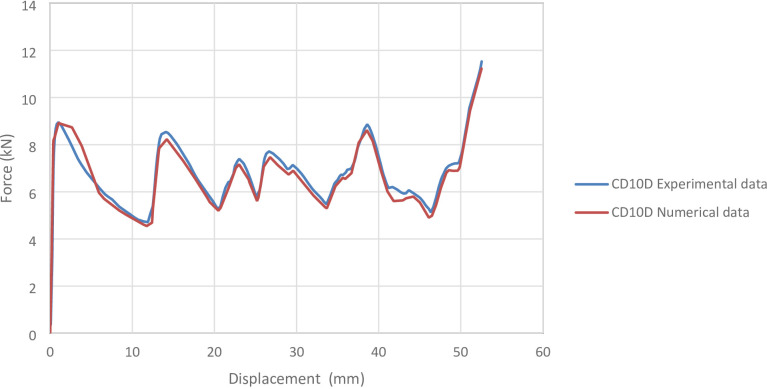
Force–displacement curve for the simulated and experimental results of CD10D specimen and model.

Figure 8 shows the axial collapse sequence of the CD10D specimen, while Fig. 9 presents the corresponding simulation stages. The experimental results reported an absorbed energy of 335.00 J, a mean crushing load of 6800.00 N, and a stroke efficiency of 0.71. The simulation predicted an absorbed energy of 335.89 J (6.24% higher), a mean crushing load of 6772.51 N (0.40% lower), and a stroke efficiency of 0.66 (6.58% lower). The simulated force–displacement curve in Fig. 10 presents the corresponding force–displacement curves for the numerical and experimental data. The model was discretized using 65,495 elements, providing a detailed representation of the axial behaviour. The underestimation of stroke efficiency is consistent with previous models and is attributed to the idealized folding mechanism in simulation, where real geometric imperfections are not fully captured. Overall, the CD10D simulation showed close agreement with the experimental results, particularly for energy absorption and mean load.

#### CD15D specimen

**Fig. 11:**
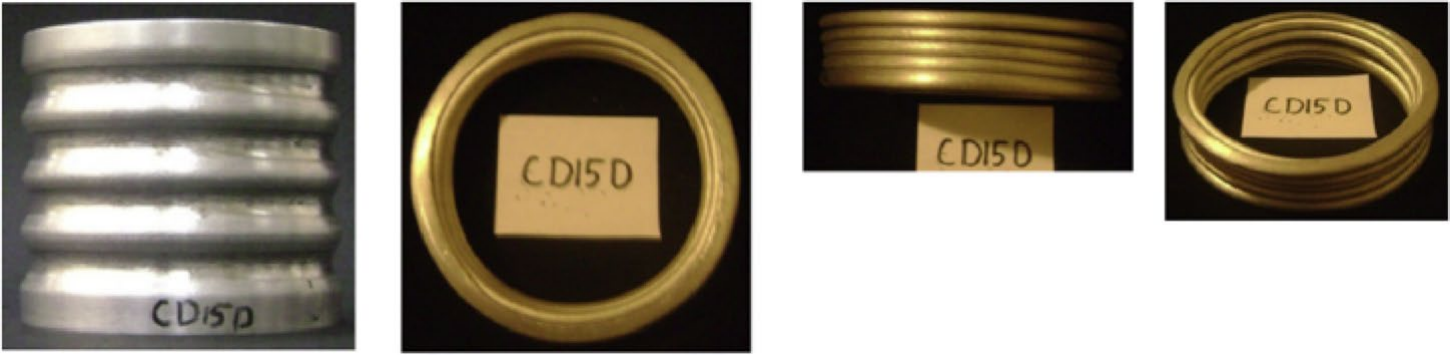
Different stages of experimental axial collapse of CD15D specimen^32^.

**Fig. 12:**
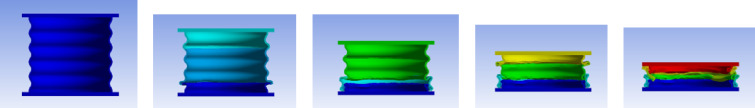
Different stages of axial collapse of specimens CD15D simulation model.

**Fig. 13:**
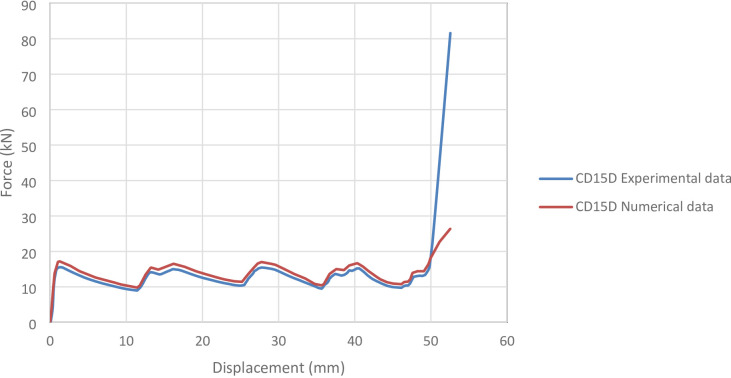
Force–displacement curve for the simulated and experimental results of CD15D specimen and model.

Figure 11 shows the axial collapse sequence of the CD15D specimen during the experiment, while Fig. 12 illustrates the simulation stages and Fig. 13 presents the corresponding force–displacement curves for the numerical and experimental data. Experimentally, the absorbed energy was 617.00 J, the mean crushing load was 12,400.00 N, and the stroke efficiency was 0.71. The simulation predicted an absorbed energy of 742.90 J (20.41% higher), a mean crushing load of 14,137.42 N (14.01% higher), and a stroke efficiency of 0.66 (7.04% lower). The model was discretized with 71,768 elements, enabling detailed tracking of deformation throughout axial loading. Although the simulation overestimated energy and load, it reproduced the overall collapse trend. The deviations are attributed to the absence of geometric imperfections, strain rate effects, and friction in the numerical setup. Overall, the CD15D simulation demonstrated reliable predictive capability, particularly for energy absorption and load response.

#### TF10C specimen

**Fig. 14:**
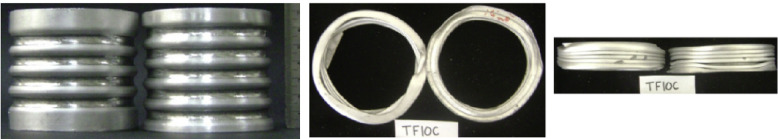
Different stages of experimental axial collapse of TF10C specimen^32^.

**Fig. 15:**
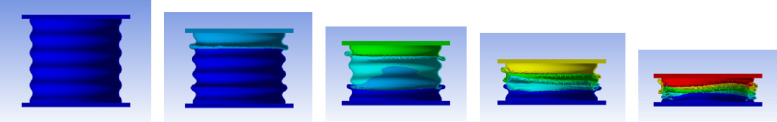
Different stages of axial collapse of specimens TF10C simulation model.

**Fig. 16:**
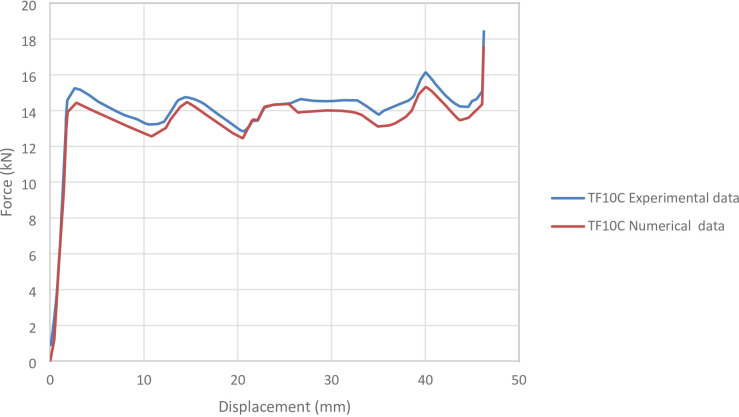
Force–displacement curve for the simulated and experimental results of TF10C specimen and model.

Figure 14 shows the axial collapse sequence of the TF10C specimen during the experiment, while Fig. 15 presents the simulation stages and Fig. 16 illustrates the corresponding force–displacement curves for the numerical and experimental data. Experimentally, the absorbed energy was 758.10 J, the mean crushing load was 14,100.00 N, and the stroke efficiency was 0.70. The simulation predicted an absorbed energy of 646.55 J (14.71% lower), a mean crushing load of 13,982.39 N (0.83% lower), and a stroke efficiency of 0.58 (18.31% lower). The model was meshed with 70,068 elements, providing sufficient detail to capture the folding mechanisms during compression. The underestimation of energy and stroke efficiency is consistent with simulation limitations, where idealized folding and early densification reduce deformation compared to real tests. Overall, the TF10C simulation showed reliable agreement with the experimental results, particularly for energy absorption and mean load.

#### TF15D specimen

**Fig. 17:**
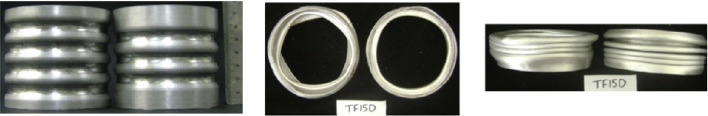
Different stages of experimental axial collapse of TF15D specimen^32^.

**Fig. 18:**
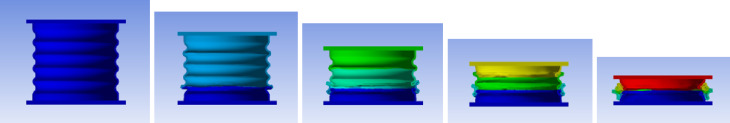
Different stages of axial collapse of specimens TF15D simulation model.

**Fig. 19:**
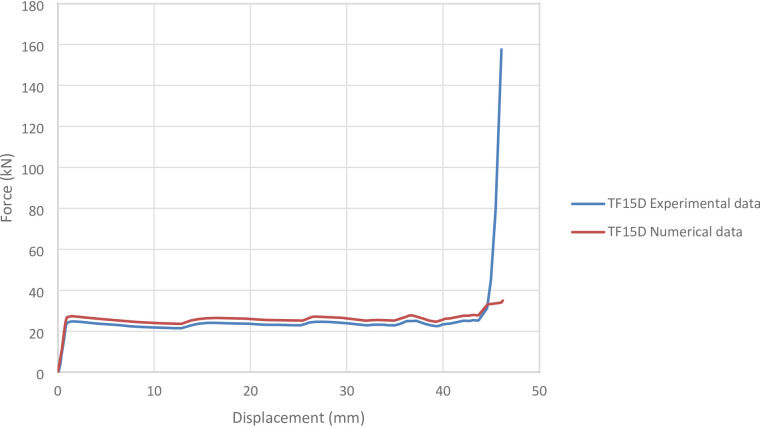
Force–displacement curve for the simulated and experimental results of TF15D specimen and model.

Figure 17 shows the axial collapse sequence of the TF15D specimen during the experiment, while Fig. 18 presents the corresponding simulation stages and Fig. 19 illustrates the force–displacement curves for the numerical and experimental data. Experimentally, the absorbed energy was 1,017.00 J, the mean crushing load was 23,000.00 N, and the stroke efficiency was 0.74. The simulation predicted an absorbed energy of 1,186.57 J (16.67% higher), a mean crushing load of 25,660.96 N (11.57% higher), and a stroke efficiency of 0.58 (21.62% lower). The model was meshed with 73,037 elements, providing sufficient refinement to capture the folding behaviour. The overestimation in energy and force is attributed to idealized simulation conditions, such as perfect geometry and symmetric deformation. Overall, the TF15D simulation reproduced the crushing response with reasonable accuracy, despite deviations in efficiency.

#### CM15 specimen

**Fig. 20:**
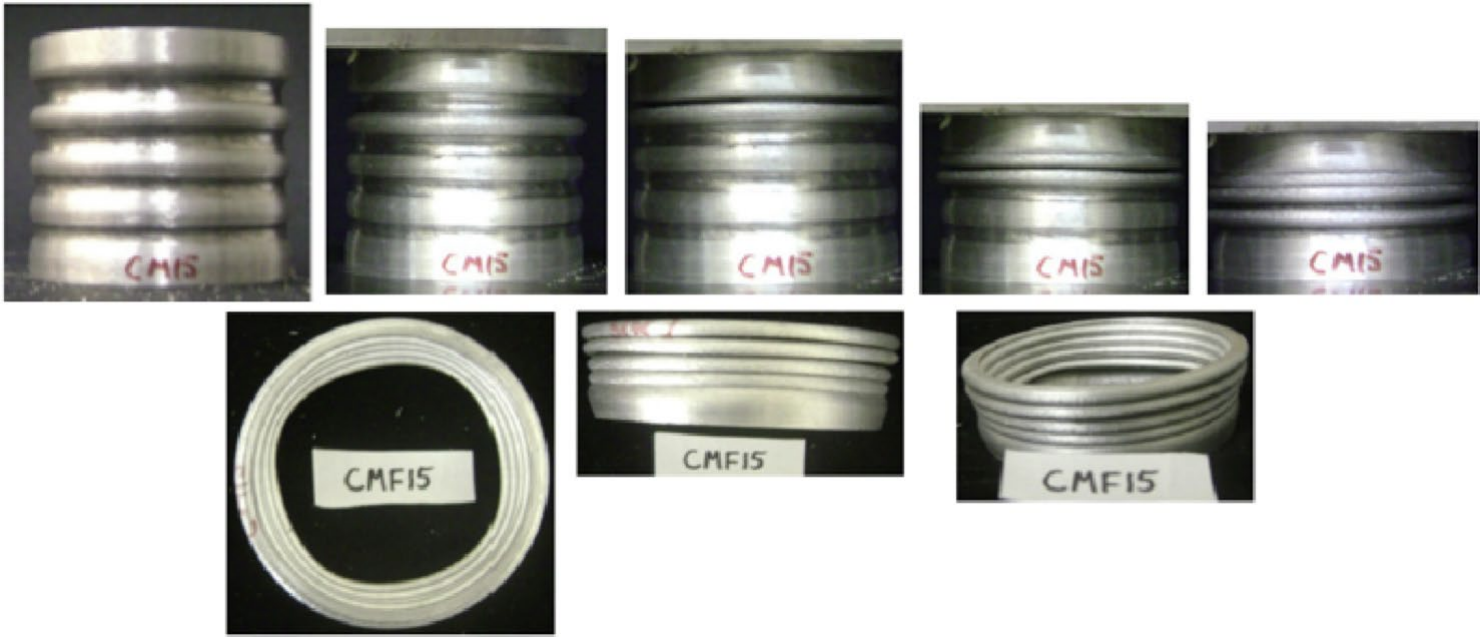
Different stages of experimental axial collapse of CM15 specimen^32^.

**Fig. 21:**
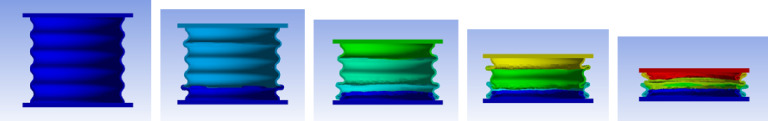
Different stages of axial collapse of specimens CM15 simulation model.

**Fig. 22:**
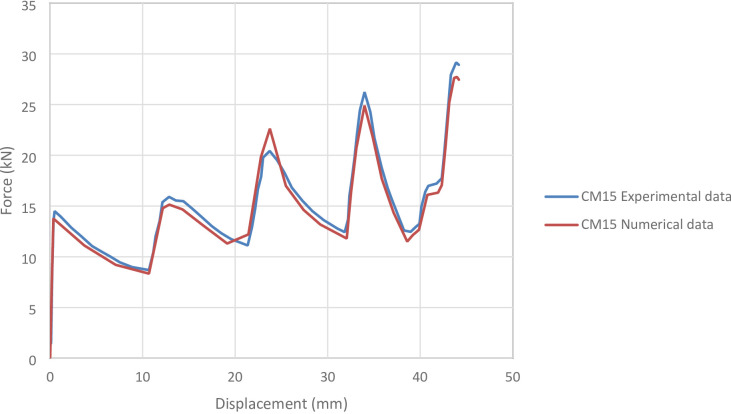
Force–displacement curve for the simulated and experimental results of CM15 specimen and model.

Figure 20 shows the axial collapse stages of the CM15 specimen during the experiment, while Fig. 21 presents the simulated deformation stages. Experimentally, the absorbed energy was 625.00 J, the mean crushing load was 14,200.00 N, and the stroke efficiency was 0.63. The simulation predicted an absorbed energy of 653.02 J (4.48% higher), a mean crushing load of 14,781.66 N (4.10% higher), and a stroke efficiency of 0.56 (11.11% lower). Figure 22 displays the corresponding force–displacement curves for the numerical and experimental data. The model was discretized with 72,936 elements, capturing the main folding behaviour during axial loading. The underestimation of stroke efficiency is attributed to idealized folding and early densification in the simulation. Overall, the CM15 simulation showed good agreement with the experimental results, particularly for energy absorption and mean load.

#### T10BF specimen

**Fig. 23:**
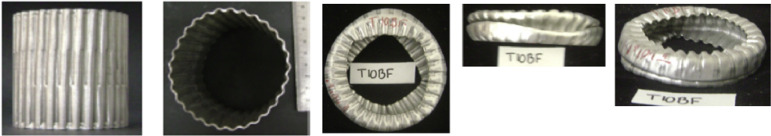
Different stages of experimental axial collapse of specimens T10BF specimen^32^.

**Fig. 24:**
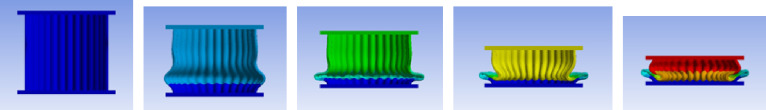
Different stages of axial collapse of specimens T10BF simulation model.

**Fig. 25:**
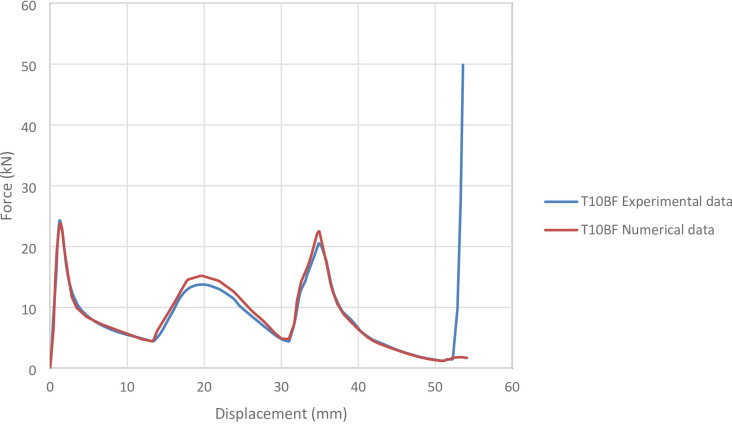
Force–displacement curve for the simulated and experimental results of T10BF specimen and model.

Figure 23 shows the axial collapse stages of the T10BF specimen during the experiment, while Fig. 24 presents the simulated deformation stages. Experimentally, the absorbed energy was 436.00 J, the mean crushing load was 10,500.00 N, and the stroke efficiency was 0.74. The simulation predicted an absorbed energy of 500.25 J (14.74% higher), a mean crushing load of 9,242.82 N (11.98% lower), and a stroke efficiency of 0.68 (8.11% lower). Figure 25 shows the corresponding force–displacement curve for the numerical and experimental data. The model was discretized with 77,713 elements, providing sufficient resolution to capture the overall crushing behaviour. Differences in energy and load are attributed to idealized folding, uniform plastic deformation, and the absence of local irregularities, strain hardening, or friction. Overall, the T10BF simulation reproduced the main crushing characteristics with reasonable accuracy.

#### T15BH specimen

**Fig. 26:**
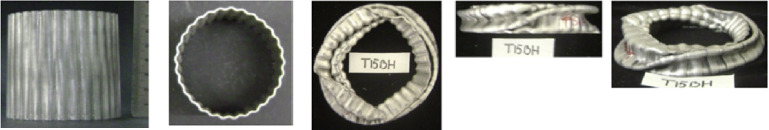
Different stages of experimental axial collapse of specimens T15BH specimen^32^.

**Fig. 27:**
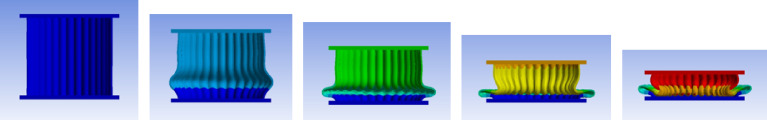
Different stages of axial collapse of specimens T15BH simulation model.

**Fig. 28:**
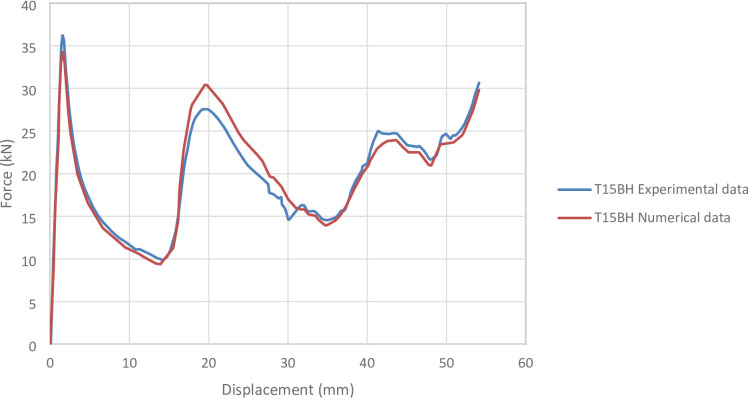
Force–displacement curve for the simulated and experimental results of T15BH specimen and model.

Figure 26 shows the axial collapse stages of the T15BH specimen during the experiment, while Fig. 27 presents the simulated deformation stages and Fig. 28 illustrates the force–displacement curves for the numerical and experimental data. Experimentally, the absorbed energy was 1,070.00 J, the mean crushing load was 20,000.00 N, and the stroke efficiency was 0.77. The simulation predicted an absorbed energy of 1,062.34 J (0.72% lower), a mean crushing load of 19,628.19 N (1.86% lower), and a stroke efficiency of 0.68 (11.69% lower). The model was discretized with 85,409 elements, providing sufficient resolution to capture the structural response during compression. The slight underestimation in stroke efficiency is attributed to the idealized, symmetric folding in the simulation. Overall, the T15BH simulation reproduced the main energy and load responses with reliable accuracy.

Table 3 presents a direct comparison of ANSYS simulation results with experimental data from the previous study, including errors for total absorbed energy, mean crushing load, and stroke efficiency. Table 4 displays the same data in an alternative format for clarity.

**Table 3:** Previous Study Experimental Result compared to Ansys Simulation Result and the error%.

Ansys Simulation Result									
Design	S101	S151	CD10D	CD15D	TF10C	TF15D	CM15	T10BF	T15BH
Total absorbed energy (J)	468.48	811.29	355.89	742.90	646.55	1,186.57	653.02	500.25	1,062.34
Mean crushing load (N)	8,640.90	14,964.00	6,772.51	14,137.42	13,982.39	25,661.00	14,781.66	9,242.82	19,628.19
SE [Stroke Efficiency]	0.68	0.68	0.66	0.66	0.58	0.58	0.56	0.68	0.68
Previous Study Experimental Result									
Design	S101	S151	CD10D	CD15D	TF10C	TF15D	CM15	T10BF	T15BH
Total absorbed energy (J)	469.00	793.00	335.00	617.00	758.10	1,017.00	625.00	436.00	1,070.00
Mean crushing load (N)	9,050.00	15,100.00	6,800.00	12,400.00	14,100.00	23,000.00	14,200.00	10,500.00	20,000.00
SE [Stroke Efficiency]	0.77	0.73	0.71	0.71	0.71	0.74	0.63	0.74	0.77
Error%									
Design	S101	S151	CD10D	CD15D	TF10C	TF15D	CM15	T10BF	T15BH
Total absorbed energy (J)	0.111%	2.307%	6.235%	20.406%	14.714%	16.674%	4.484%	14.736%	0.716%
Mean crushing load (N)	4.520%	0.901%	0.404%	14.011%	0.834%	11.570%	4.096%	11.973%	1.859%
SE [Stroke Efficiency]	11.433%	6.580%	6.903%	6.903%	18.079%	21.400%	11.794%	8.001%	11.585%

**Table 4:** Previous Study Experimental Result compared to Ansys Simulation Result and the error%.

Design	Total absorbed energy (J) Simulation	Total absorbed energy (J) Experimental	Total absorbed energy (J) Error%	Mean crushing load (*N*) Simulation	Mean crushing load (*N*) Experimental	Mean crushing load (*N*) Error	SE [Stroke Efficiency] Simulation	SE [Stroke Efficiency] Experimental	SE [Stroke Efficiency] Error
S101	468.48	469.00	0.00	8,640.90	9,050.00	0.05	0.68	0.77	0.11
S151	811.33	793.00	0.02	14,964.68	15,100.00	0.01	0.68	0.73	0.07
CD10D	355.89	335.00	0.06	6,772.51	6,800.00	0.00	0.66	0.71	0.07
CD15D	742.90	617.00	0.20	14,137.42	12,400.00	0.14	0.66	0.71	0.07
TF10C	646.55	758.10	0.15	13,982.39	14,100.00	0.01	0.58	0.71	0.18
TF15D	1,186.57	1,017.00	0.17	25,660.96	23,000.00	0.12	0.58	0.74	0.21
CM15	653.02	625.00	0.04	14,781.66	14,200.00	0.04	0.56	0.63	0.12
T10BF	500.25	436.00	0.15	9,242.82	10,500.00	0.12	0.68	0.74	0.08
T15BH	1,062.34	1,070.00	0.01	19,628.19	20,000.00	0.02	0.68	0.77	0.12

The comparison between experimental data and simulation results indicates satisfactory agreement, demonstrating the reliability and predictive capability of the developed finite element models across nine designs, ranging from simple cylindrical tubes to complex corrugated and laterally modified geometries. Given the accuracy and consistency of these models, the same simulation setup will be applied in the next phase, which investigates the effect of corrugation inclination angle on the crashworthiness of cylindrical energy absorbers.

### Model-by-model discussion for proposed design

Crashworthiness remains a critical factor in the design of structural energy absorbers for automotive, aerospace, and protective applications. One common approach to improve energy absorption is the introduction of geometric features, such as corrugation patterns, into cylindrical tubes. Studies have shown that corrugations can influence deformation behaviour, delay densification, and enhance energy dissipation. Rather than focusing on a single corrugation type, this study builds on the broader understanding that such modifications improve performance.

This section presents a numerical investigation of how corrugation inclination angle affects the crashworthiness of cylindrical energy absorbers. A smooth, non-corrugated tube serves as a baseline, while inclined corrugated models with angles of 0°, 15°, 30°, 45°, 60°, and 90° are analysed. These variations explore how tilting the corrugation profile affects folding patterns, local strain distribution, and load transfer during axial compression. The hypothesis is that changing the corrugation orientation will shift the absorber from symmetric folding toward more complex deformation modes, potentially altering energy dissipation.

To provide a comprehensive assessment, additional crashworthiness parameters are introduced alongside Total Absorbed Energy (TAE), Mean Crushing Load (MCL), and Stroke Efficiency (SE):

- Crushing Length: Effective displacement over which the absorber undergoes meaningful deformation.
- Specific Energy Absorption (SEA): Energy absorbed per unit mass (J/kg), enabling weight-neutral comparisons.
- Initial Peak Force (IPF): Highest load at the onset of compression, relevant for minimizing impact shock.
- Crushing Force Efficiency (CFE): Ratio of mean load to peak load, reflecting stability of energy absorption.

All simulations use the same boundary and loading conditions as in the previous section. TAE is calculated from the area under the ANSYS-generated force–displacement curve, exported to Excel and processed using the Trapezoidal Rule. MCL is obtained by dividing TAE by maximum displacement, IPF is taken from the initial peak on the curve, and CFE is computed as the ratio of mean load to peak force.

Slight differences in mass among designs are accounted for in SEA calculations. These variations also influence meshing requirements, as more complex geometries require finer element distributions to capture deformation accurately.

#### No corrugation model

**Fig. 29:**
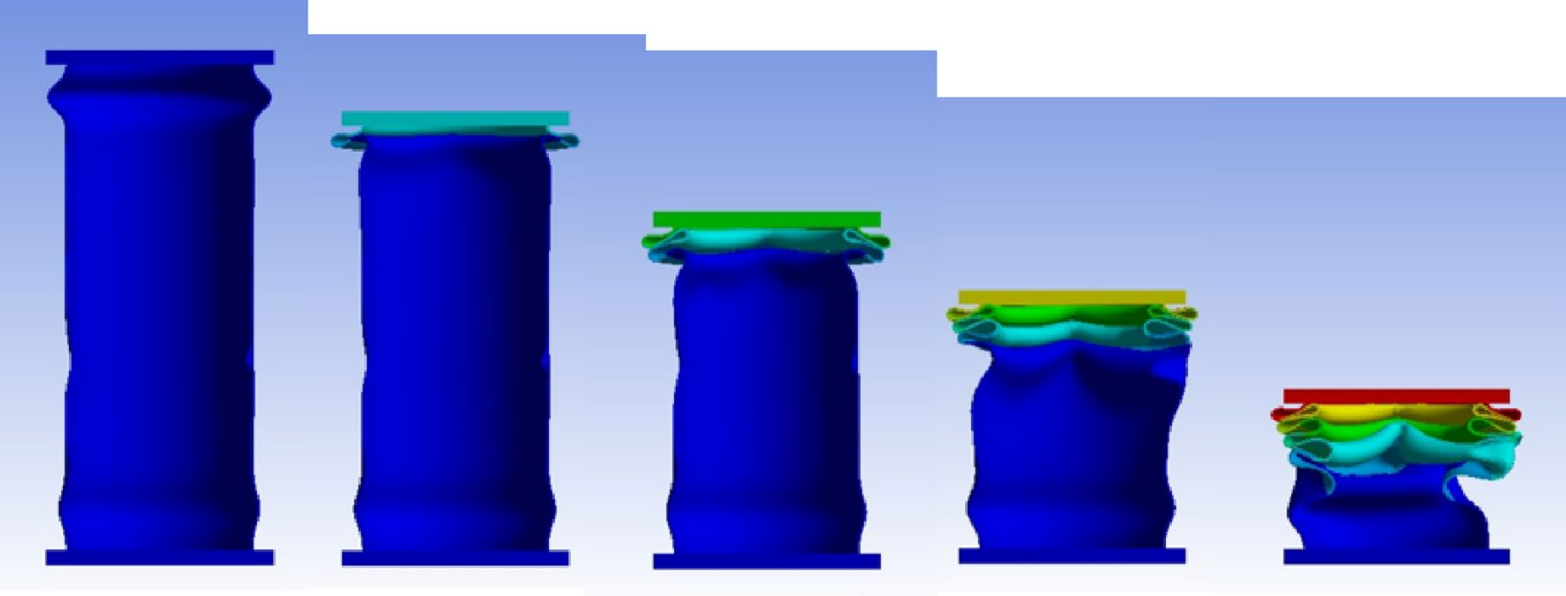
Different stages of axial collapse of the no corrugation cylinder simulation model.

**Fig. 30:**
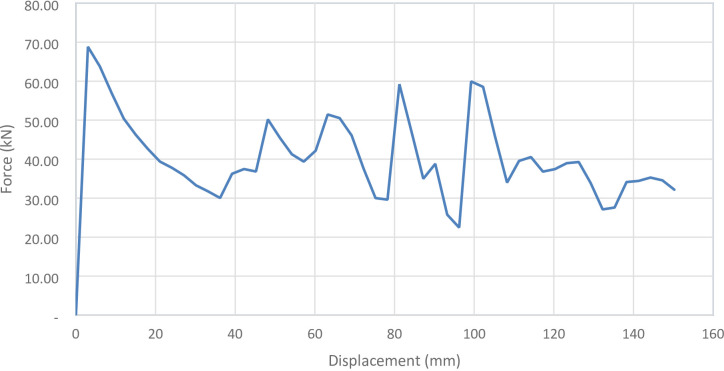
Force–displacement curve for the no corrugation cylinder simulated results generated by Excel.

Figure 29 shows the axial collapse stages of the smooth cylindrical tube, while Fig. 30 presents the corresponding simulation force–displacement curve. Serving as the baseline model, the tube deformed through a classic axisymmetric folding pattern, forming rings progressively along its length. This behaviour enabled consistent and stable energy absorption. The simulation predicted a Total Absorbed Energy (TAE) of 6049.32 J, a Mean Crushing Load (MCL) of 40,261.69 N, and a Specific Energy Absorption (SEA) of 45,783.09 J/kg for a total mass of 0.13213 kg. The Initial Peak Force (IPF) was 68,817.00 N, and the Crushing Force Efficiency (CFE) was 58.51%. The model was meshed with 164,206 elements, allowing precise tracking of folding sequences and local strain behaviour.

#### 0° inclination angle model

**Fig. 31:**
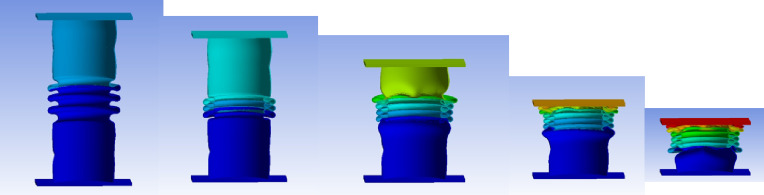
Different stages of axial collapse of the 0 degrees (horizontal) corrugated cylinder simulation model.

**Fig. 32:**
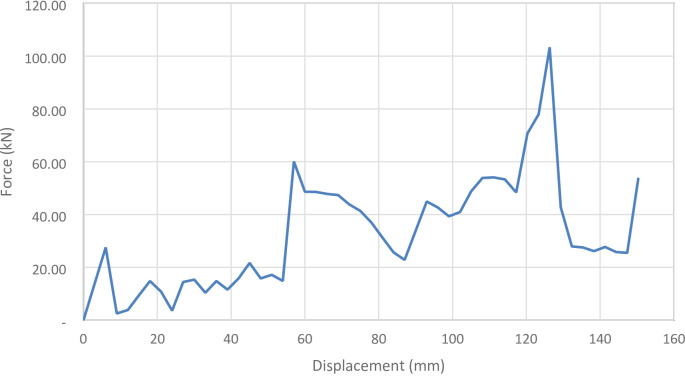
Force–displacement curve for the 0 degrees (horizontal) corrugated cylinder simulated results generated by Excel.

Figure 31 shows the axial collapse stages of the 0° inclination corrugated tube, while Fig. 32 presents the corresponding force–displacement curve. The straight corrugations, aligned perpendicular to the tube axis, promoted a regular folding pattern during compression. The simulation predicted a Total Absorbed Energy (TAE) of 4921.92 J, a Mean Crushing Load (MCL) of 32,740.77 N, and a crushing length of 150.33 mm, resulting in a stroke efficiency of 72.27%. For a mass of 0.15538 kg, the Specific Energy Absorption (SEA) was 31,676.02 J/kg. The Initial Peak Force (IPF) reached 27,555 N, and the Crushing Force Efficiency (CFE) was 118.82%. The model was meshed with 189,686 elements, allowing accurate tracking of folding and local strain behavior.

#### 15° inclination angle model

**Fig. 33:**
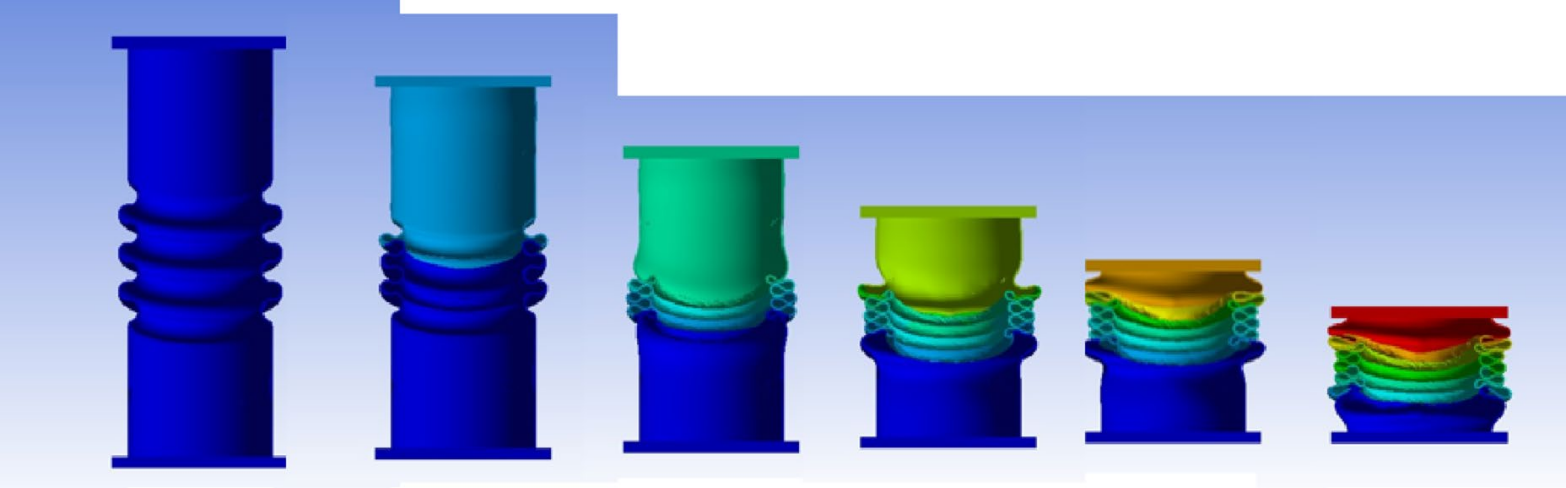
: Different stages of axial collapse of the 15 degrees (measured from horizontal line) corrugated cylinder simulation model.

**Fig. 34:**
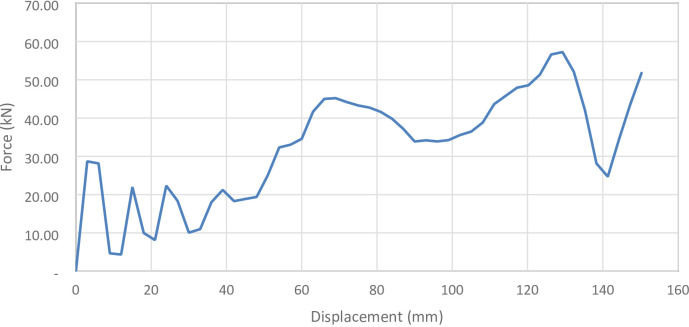
Force–displacement curve for the 15 degrees (measured from horizontal line) corrugated cylinder simulated results generated by Excel.

Figure 33 shows the axial collapse stages of the 15° inclination corrugated tube, while Fig. 34 presents the corresponding force–displacement curve. The shallow tilt in the corrugation profile produced stable deformation, though energy absorption and load resistance were slightly lower than more optimized folding patterns. The simulation predicted a Total Absorbed Energy (TAE) of 4838.80 J, a Mean Crushing Load (MCL) of 32,194.27 N, and a crushing length of 150.30 mm, resulting in a stroke efficiency of 72.26%. For a mass of 0.15737 kg, the Specific Energy Absorption (SEA) was 30,767.46 J/kg. The Initial Peak Force (IPF) was 28,686.00 N, and the Crushing Force Efficiency (CFE) was 112.23%. The model was meshed with 187,596 elements, capturing folding and local strain behaviour accurately.

#### 30° inclination angle model

**Fig. 35:**
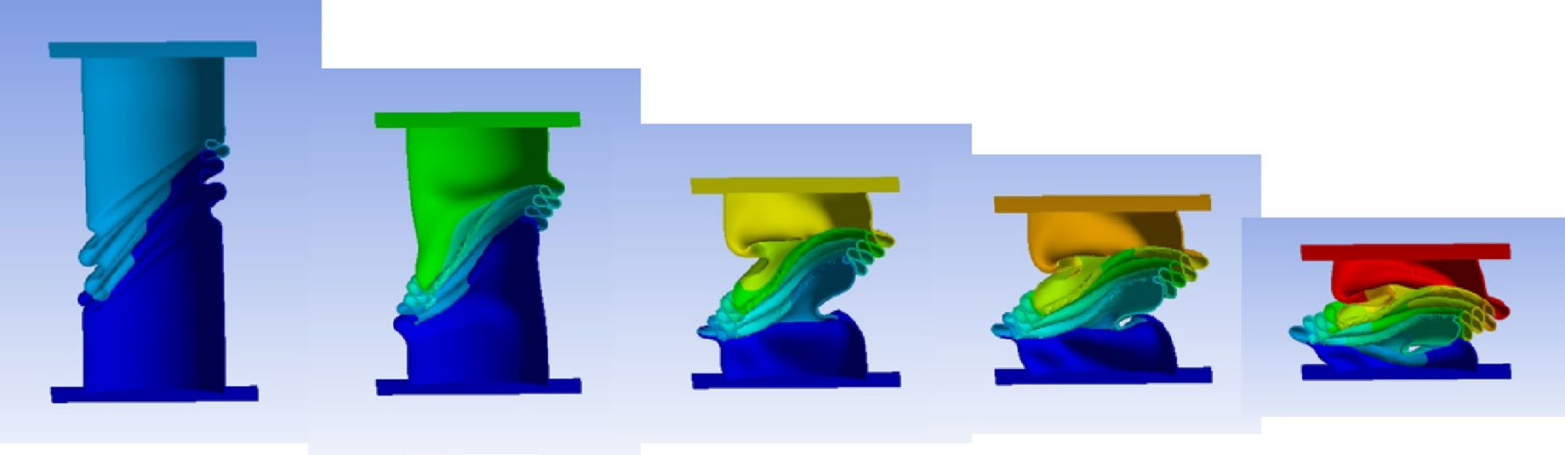
Different stages of axial collapse of the 30 degrees (measured from horizontal line) corrugated cylinder simulation model.

**Fig. 36:**
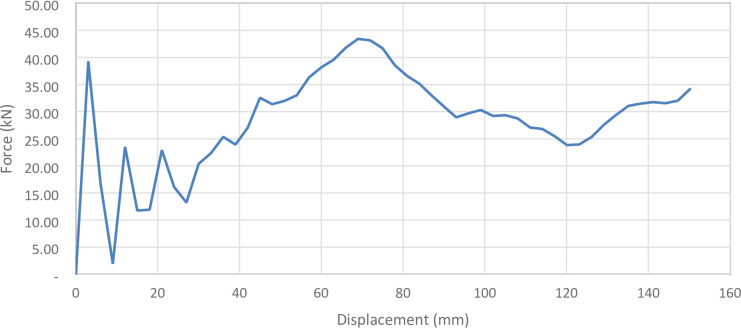
Force–displacement curve for the 30 degrees (measured from horizontal line) corrugated cylinder simulated results generated by Excel.

Figure 35 shows the axial collapse stages of the 30° inclined corrugated tube, while Fig. 36 presents the corresponding force–displacement curve. The 30° inclination introduces a deviation from the axial direction, affecting folding patterns. The simulation predicted a Total Absorbed Energy (TAE) of 4278.42 J, a Mean Crushing Load (MCL) of 28,484.81 N, and a crushing length of 150.2 mm, resulting in a stroke efficiency of 72.21%. For a mass of 0.16247 kg, the Specific Energy Absorption (SEA) was 26,333.34 J/kg. The Initial Peak Force (IPF) reached 39,296 N, and the Crushing Force Efficiency (CFE) was 72.49%. The model was meshed with 188,876 elements, capturing the folding and local strain behavior accurately.

#### 45° inclination angle model

**Fig. 37:**
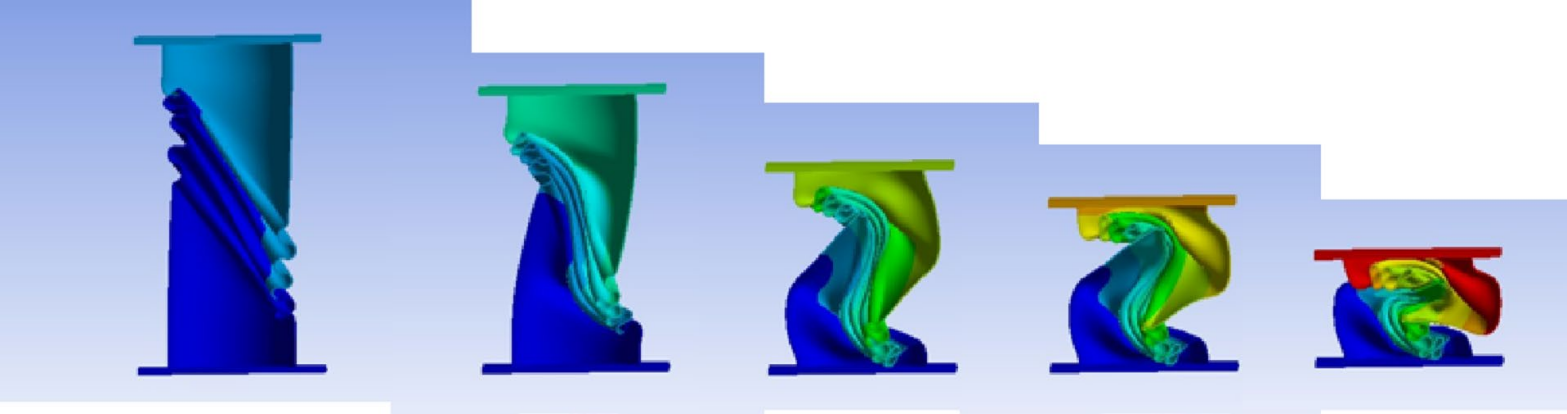
Different stages of axial collapse of the 45 degrees (measured from horizontal line) corrugated cylinder simulation model.

**Fig. 38:**
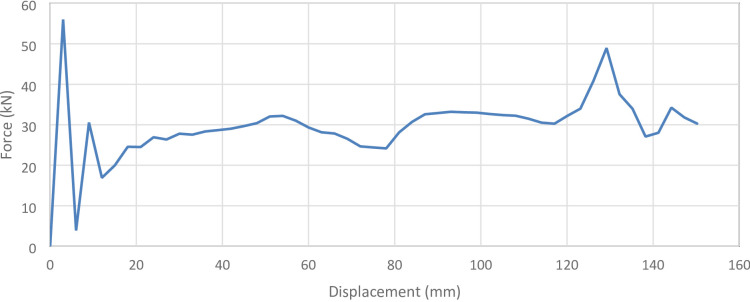
Force–displacement curve for the 45 degrees (measured from horizontal line) corrugated cylinder simulated results generated by Excel.

Figure 37 shows the axial collapse stages of the 45° inclined corrugated tube, while Fig. 38 presents the corresponding force–displacement curve. The simulation predicted a Total Absorbed Energy (TAE) of 4473.99 J, a Mean Crushing Load (MCL) of 29,780.91 N, and a crushing length of 150.23 mm, resulting in a stroke efficiency of 72.23%. For a mass of 0.16633 kg, the Specific Energy Absorption (SEA) was 26,897.77 J/kg. The Initial Peak Force (IPF) reached 56,000 N, and the Crushing Force Efficiency (CFE) was 53.18%, indicating strong initial resistance followed by a reduction in sustained load. The deformation remained orderly, though energy absorption was limited by early softening and reduced plastic hinge development. The model was meshed with 194,316 elements, allowing accurate capture of folding and local strain behavior.

#### 60° inclination angle model

**Fig. 39:**
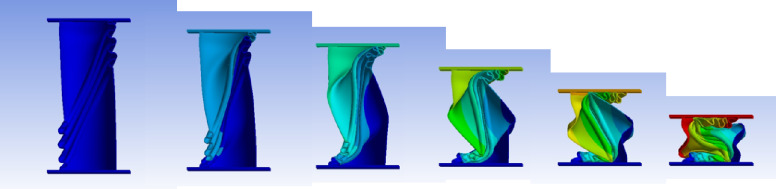
Different stages of axial collapse of the 60 degrees (measured from horizontal line) corrugated cylinder simulation model.

**Fig. 40:**
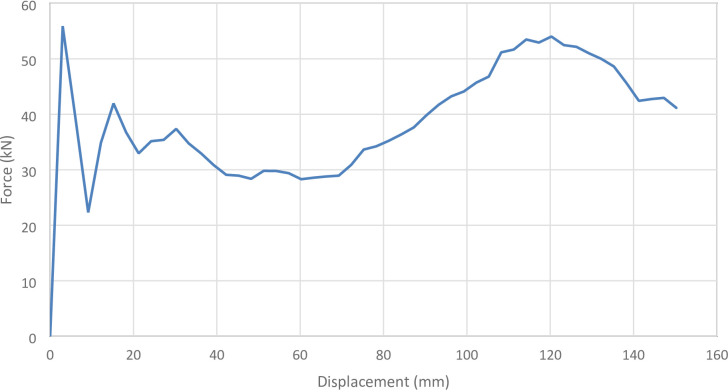
Force –displacement curve for the 60 degrees (measured from horizontal line) corrugated cylinder simulated results generated by Excel.

Figure 39 shows the axial collapse stages of the 60° inclined corrugated tube, while Fig. 40 presents the corresponding force–displacement curve. The steep 60° inclination promotes a complex folding pattern with inclined and twisting deformations. The simulation predicted a Total Absorbed Energy (TAE) of 5835.34 J, a Mean Crushing Load (MCL) of 38,834.96 N, and a crushing length of 150.26 mm, resulting in a stroke efficiency of 72.24%. For a mass of 0.18537 kg, the Specific Energy Absorption (SEA) was 31,478.76 J/kg. The Initial Peak Force (IPF) reached 55,893 N, and the Crushing Force Efficiency (CFE) was 69.48%, indicating strong initial stiffness with moderately stable load during crushing. The model was meshed with 220,243 elements, allowing accurate capture of folding and local strain behavior.

#### 90° inclination angle model

**Fig. 41:**
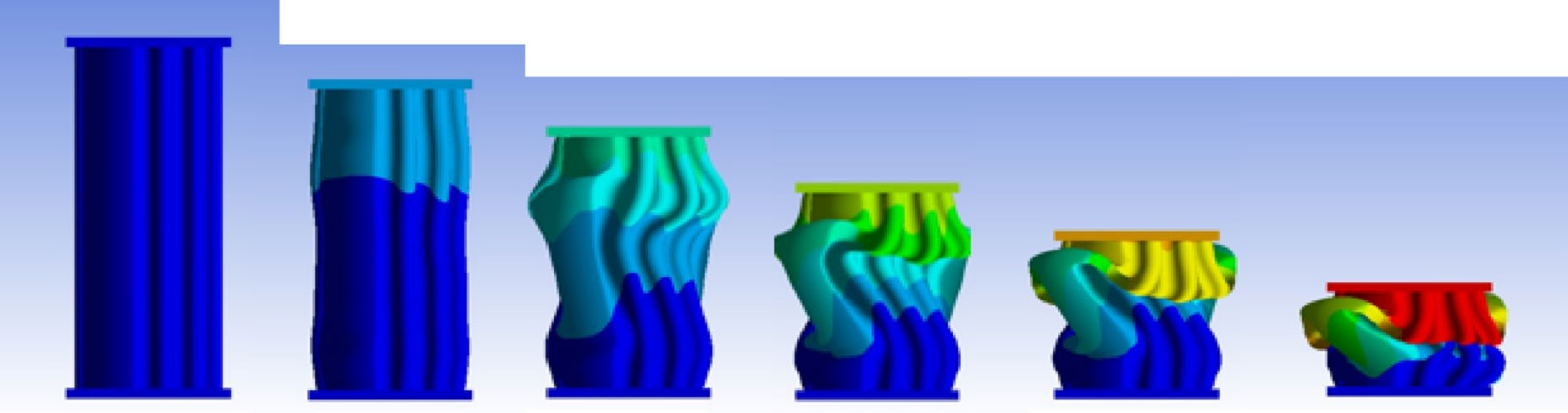
Different stages of axial collapse of the 90 degrees (Vertical) corrugated cylinder simulation model.

**Fig. 42:**
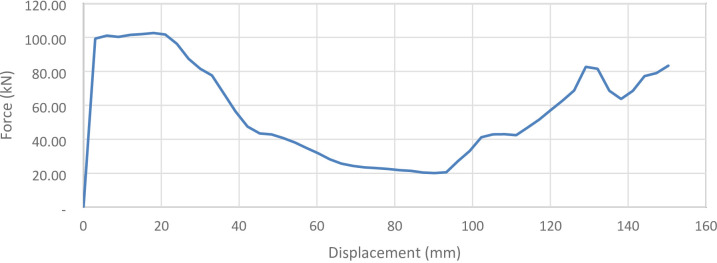
Force–displacement curve for the 90 degrees (vertical) corrugated cylinder simulated results generated by Excel.

Figure 41 shows the axial collapse stages of the 90° inclined corrugated tube, while Fig. 42 presents the corresponding force–displacement curve. With grooves fully parallel to the loading direction, this design exhibited a distinct collapse mode. The simulation predicted a Total Absorbed Energy (TAE) of 8382.55 J, a Mean Crushing Load (MCL) of 55,757.27 N, and a crushing length of 150.34 mm, resulting in a stroke efficiency of 72.28%. The Initial Peak Force (IPF) reached 102,570 N, and the Crushing Force Efficiency (CFE) was 54.36%. For a mass of 0.17549 kg, the Specific Energy Absorption (SEA) was 47,810.12 J/kg, the highest among all models. The high IPF indicates strong initial stiffness, while the lower CFE reflects a force drop-off after the peak. The model was meshed with 215,366 elements, capturing folding patterns and local strain behaviour accurately.

**Fig. 43:**
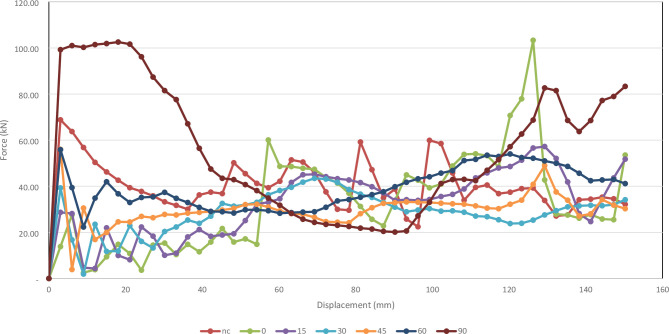
All the force-displacement curves for the not corrugated, 0°, 15°, 30°, 45°, 60°, and 90°.

Figure 43 below shows all of the different corrugation inclination angles force-displacement curves.

#### Comprehensive summary and analysis

Varying corrugation inclination angles from smooth (no corrugation) to 0°, 15°, 30°, 45°, 60°, and 90 significantly affects the crashworthiness of thin-walled cylindrical absorbers. Key performance indicators include Total Absorbed Energy (TAE), Mean Crushing Load (MCL), Specific Energy Absorption (SEA), Initial Peak Force (IPF), Crushing Force Efficiency (CFE), and Stroke Efficiency (SE). Performance does not improve linearly with inclination. The following analysis summarizes the numerical results and interprets them using principles of plastic collapse and shell mechanics.

##### Total Absorbed Energy (TAE)

**Fig. 44:**
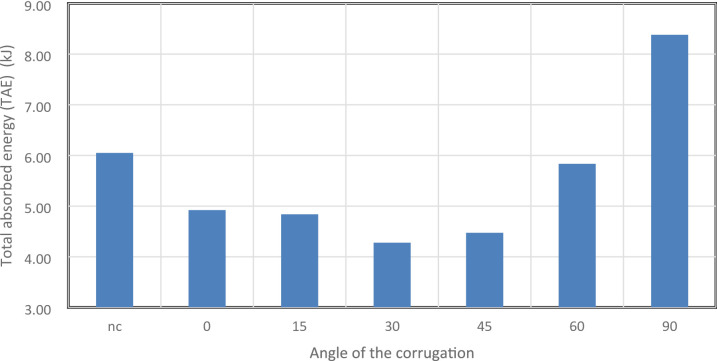
This graph shows the Total absorbed energy (TAE) in kJ for the different corrugation angels as well as the not corrugated (nc).

Figure 44 shows TAE for all models, representing the total energy absorbed through plastic deformation. The 90° model achieved the highest TAE at 8,382.55 J, followed by the 60° model at 5,835.34 J, an 18.6% increase over the 0° baseline (4,921.92 J). In contrast, the 30° and 45° models recorded lower energy absorption (4,278.42 J and 4,473.99 J), likely due to less stable or premature folding. The 15° design also underperformed relative to the 0° model, and all corrugated designs up to 45° absorbed less energy than the smooth tube (6,049.32 J). These results indicate that only steep inclinations promote favourable multi-axial deformation modes that enhance energy dissipation. Shallow and mid-range angles tend to disrupt stable folding, whereas high angles introduce new folding paths that increase total energy absorption.

##### Mean Crushing Load (MCL)

**Fig. 45:**
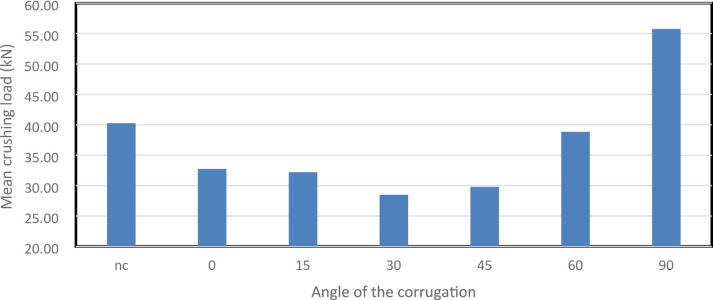
This graph shows the Mean crushing load (MCL) in kN for the different corrugation angels as well as the not corrugated (nc).

Figure 45 shows the MCL values for the smooth and corrugated tubes. MCL represents the average structural resistance during crushing. The 90° model reached the highest MCL at 55,757.27 N, followed by the 60° and smooth tubes at 38,834.96 N and 40,261.69 N, respectively. The 0° configuration recorded 32,740.77 N, while the 30° and 45° designs dropped to 28,484.81 N and 29,780.91 N. Mid-range angles appear to reduce axial stiffness, likely due to uneven stress distribution. Higher inclinations improve MCL by redistributing loads more effectively, acting as internal bracing that delays collapse.

##### Specific Energy Absorption (SEA)

**Fig. 46:**
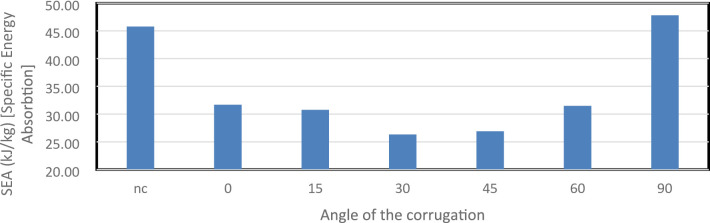
This graph shows the Specific Energy Absorption (SEA) in kJ/kg for the different corrugation angels as well as the not corrugated (nc).

Figure 46 presents SEA values for all models, indicating energy absorbed per unit mass. The 90° model achieved the highest SEA at 47,810.12 J/kg, followed by the smooth tube at 45,783.09 J/kg. The 0° and 60° designs were similar, with 31,676.02 J/kg and 31,478.76 J/kg, while the 30° and 45° models recorded the lowest SEAs at 26,333.34 J/kg and 26,897.77 J/kg. Higher inclinations improve energy absorption efficiency per kilogram despite slightly increased mass, whereas mid-range angles underperform due to unstable folding and less efficient deformation.

##### Initial Peak Force (IPF)

**Fig. 47:**
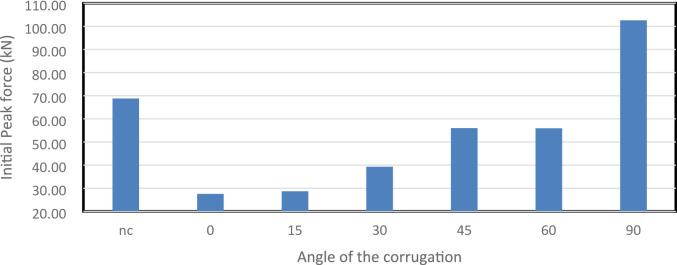
This graph shows the Initial Peak force (IPF) in kN for the different corrugation angels as well as the not corrugated (nc).

Figure 47 shows IPF values for all models, representing the maximum force at the start of deformation. The 0° model had the lowest IPF at 27,555 N, providing a gentle initial response. The 15° and 30° designs increased moderately to 28,686 N and 39,296 N, while the 45° and 60° models reached 56,000 N and 55,893 N. The 90° model peaked at 102,570 N, the highest value. These results highlight a trade-off: higher inclinations enhance structural resistance but generate larger initial force spikes, which may be undesirable in applications sensitive to shock loads.

##### Crushing Force Efficiency (CFE)

CFE measures the consistency of force during compression by comparing mean crushing force to the peak force. The 0° model achieved the highest CFE at 118.82%, followed by the 15° and 30° designs at 112.23% and 72.49%. The 45°, 60°, and 90° models recorded lower CFEs of 53.18%, 69.48%, and 54.36%, respectively. Lower CFE in high-angle models reflects a rapid drop in force after the peak, indicating a transition from stiff to soft *behaviour*. While these designs absorb more total energy, their force distribution is less uniform, potentially leading to less predictable responses under dynamic loading.

##### Deformation behaviour and stability

Visual inspection showed that the 0° model maintained stable, symmetric folds, while the 15° design introduced minor asymmetry but remained consistent. The 30° and 45° models exhibited irregular, asymmetric folding, indicating mechanical instability. The 60° and 90° designs recovered stability through shear-dominated collapse modes that distributed deformation over a wider area. These observations align with the numerical results: stable folding supports efficient energy absorption, mid-range inclinations disrupt this pattern, and high-angle designs, while less predictable, often achieve higher total energy absorption through alternative deformation mechanisms.

##### Design implications and recommendations

Each configuration presents trade-offs. The 0° model offers low IPF, high CFE, and consistent folding, making it suitable for safety-critical applications. The 60° and 90° designs maximize energy absorption and mass efficiency but generate high initial forces, making them more appropriate for industrial, aerospace, or military structures where energy dissipation is prioritized over occupant comfort. The 30° and 45° models showed weak and inconsistent performance and are not recommended unless modifications, such as variable thickness or hybrid materials, can improve stability. The optimal design depends on the specific crash scenario. Understanding these trade-offs allows engineers to select geometries tailored to application requirements. Table 5 summarizes all crashworthiness metrics across the models.

**Table 5:** Proposed Study Simulation Result.

Angle	No corrugations	0	15	30	45	60	90
Total absorbed energy (TAE) (J)	6,049.32	4,921.92	4,866.87	4,278.42	4,473.99	5,835.34	8,382.55
Mean crushing load (N)	40,261.69	32,740.77	32,381.03	28,484.81	29,780.91	38,834.96	55,757.27
Crushing Length (mm)	150.25	150.33	150.30	150.20	150.23	150.26	150.34
Tube Length (mm)	208.00	208.00	208.00	208.00	208.00	208.00	208.00
Mass (kg)	0.13	0.16	0.16	0.16	0.17	0.19	0.18
SE [Stroke Efficiency]	72.24%	72.27%	72.26%	72.21%	72.23%	72.24%	72.28%
SEA (J/kg) [Specific Energy Absorption]	45,783.09	31,676.02	30,945.94	26,333.34	26,897.77	31,478.76	47,810.12
Initial Peak force (N)	68,817.00	27,555.00	28,686.00	39,296.00	56,000.00	55,893.00	102,570.00
Crushing Force Efficiency [CFE]	58.51%	118.82%	112.88%	72.49%	53.18%	69.48%	54.36%

## Conclusion

This study presented a numerical investigation on the effect of corrugation inclination on the crashworthiness of thin-walled cylindrical energy absorbers under quasi-static axial loading. Seven geometries were analysed: a smooth tube and six corrugated tubes with angles of 0°, 15°, 30°, 45°, 60°, and 90°. Key metrics—Total Absorbed Energy (TAE), Mean Crushing Load (MCL), Stroke Efficiency (SE), Specific Energy Absorption (SEA), Initial Peak Force (IPF), and Crushing Force Efficiency (CFE)—were used to evaluate energy dissipation and structural response.

Results showed a non-linear effect of inclination. The smooth tube absorbed 6,049.32 J through stable, concertina folding. Low-angle models (0° and 15°) recorded lower TAE (4,921.92 J and 4,838.80 J) due to early folding and interrupted hinge formation. Mid-range angles (30° and 45°) performed worse (4,278.42 J and 4,473.99 J) because of irregular folding and asymmetric stress paths. High-angle models improved performance, with the 60° tube absorbing 5,835.34 J and the 90° tube reaching 8,382.55 J, a 70.3% increase over the 0° design. Steep corrugations acted as built-in stiffeners, delaying folding and promoting uniform, shear-dominated deformation.

IPF trends showed the opposite pattern: the 0° and 15° models had the lowest peaks (27,555 N and 28,686 N), while 45°, 60°, and 90° models recorded much higher IPFs (56,000 N, 55,893 N, 102,570 N), reflecting higher initial stiffness and delayed yielding. SEA results mirrored TAE trends, with the 90° model achieving the highest efficiency (47,810.12 J/kg) and mid-range angles the lowest. CFE analysis indicated that the 0° model maintained the most consistent force (118.82%), whereas high-angle models, especially 90°, showed sharp drops in force after the peak (54.36%), indicating less uniform energy dissipation.

Overall, inclination angle strongly affects performance. Low inclinations favor low IPF and consistent deformation, mid-range angles reduce stability and energy absorption, and high inclinations maximize TAE and SEA but increase initial forces and reduce CFE. Design choice should reflect the specific application requirements, balancing energy absorption, force consistency, and peak load.

Future work should include experimental validation, dynamic loading tests, material and thickness variations, and advanced geometries such as graded or multi-stage corrugations. These steps will support the development of energy absorbers that optimize the balance between energy dissipation, structural stability, and peak force management.

## Data Availability

The datasets generated and/or analyzed during the current study are not publicly available due to ongoing data processing and analysis. However, the data will be made available upon reasonable request from the corresponding author once the study is concluded.
